# Beneficial sterols in selected edible insects and their associated antibacterial activities

**DOI:** 10.1038/s41598-023-37905-4

**Published:** 2023-07-04

**Authors:** Cynthia Muhavi Mudalungu, Hosea Oginda Mokaya, Chrysantus Mbi Tanga

**Affiliations:** International Centre of Insect Physiology and Ecology (ICIPE), P.O. Box 30772, Nairobi, 00100 Kenya

**Keywords:** Chemical biology, Chemistry

## Abstract

Edible insects are increasingly gaining popularity as research reveals multiple benefits. However, the rediscovery of natural products from insects as medicinal agents has received limited attention. This study aimed at evaluating the diversity of sterols in extracts of nine edible insects and potential antibacterial activities. Dichloromethane extracts of these insects were analyzed using gas chromatography–mass spectrometry to identify important sterols, followed by evaluation of their anti-bacterial activities. Nineteen sterols were identified with the highest recorded in African fruit beetle [*Pachnoda sinuata* (47.37%)], crickets [*Gryllus bimaculatus* (36.84%) and *Scapsipedus icipe* (31.58%)]. Cholesterol was the most prevalent, except in black soldier fly (*Hermetia illucens*). Bioactivity revealed *S. icipe* as the most potent extract against *Escherichia coli* and *Bacillus subtilis* whereas *G. bimaculatus* was highest against Methicillin-susceptible *Staphylococcus aureus* 25923. These findings unravels the diversity of sterols in edible insects and their possible application in food, pharmaceutical and cosmetic industries.

## Introduction

Edible insects have long been regarded as nutritious food items as well as wholesome elements in several meals and traditional subsistence components. They contain a variety of nutrients representing good sources of proteins, fat, minerals, vitamins, and energy as well other chemical components^1–3^. Apart from them offering a good source of nutrients, they also appear to have health benefits not only for humans and animals, but also for plants utilizing left over substrate for growth^4^. Sterols which are among their present chemical components are amphipathic compounds possessing a characteristic perhydro-1,2-cyclopentanophenanthrene ring skeleton and an attached side chain derived from a highly conserved mevalonate pathway^5^. All eukaryotes require these sterols for critical cellular functions like balancing membrane fluidity, phagocytosis, stress tolerance, and cell signaling^6–10^. Other novel biochemical reactions they portray have also been discovered to include new regulatory mechanisms that provide important insights into sterol transportation^5,11,12^.

Cholesterol as an example is ubiquitous in the animal kingdom dating back to the early 1900s, whereas its isomeric forms, known as "cholesterol bodies", have been commonly found in the plant kingdom^13^. Their main distinction lies in the side chain, which has varying degrees of substitution and unsaturation. So far, at least 250 sterols and their related steranes have been documented from prokaryotes, eukaryotes, and other hydrocarbon source rocks^14,15^. Nonetheless, there is a scarcity of information on insect-related sterols especially from edible ones.

The consumption of food containing phytosterols has a variety of biological benefits on the human body. This includes the reduction of intestinal cholesterol absorption, resulting in lowered blood serum low-density lipoprotein cholesterol (LDL-C) levels, thus reducing the risk of cardiovascular diseases^16^. Aside from that, phytosterols have beneficial effects on non-lipid variables such as inflammation^17^, oxidative stress markers, coagulation parameter and endothelial function modulation^18,19^, anticancer^20,21^, and immuno-regulatory effects^22^. It is also noted that higher plants, algae, fungi, and vertebrates synthesize most sterols. On the other hand, insects, which account for over 80% of all animal species, are unable to produce sterols on their own and are mainly acquired in dietary forms^23^. However, cholesterol as the dominant tissue sterol for most insect herbivores is produced by metabolizing phytosterols, since they grow on a mixed-sterol diet, with varied metabolic abilities depending on the types and ratio of dietary sterols^24^. The diversity of phytosterols determines the distinct functions of vital activities. For instance, the biological variation of *β*-sitosterol and campesterol have been associated as a measure of cholesterol absorption, whereas lathosterol is a biomarker for cholesterol production^25^.

Recently, studies have focused on the possible benefits and mechanisms of action of phytosterols on cancer, which show the reduction of incidences of lung, stomach, ovarian, and breast tumors. Phytosterols also appear to decrease carcinogen production, cancer cell proliferation, angiogenesis, invasion, and metastasis, increase increasing cancer cell apoptosis, through a variety of pathways^20^. Moreover, in the past year Menni et al. identified sterols from the plant *Anabasis articulata* (Forssk.) Moq. (Chenopodiaceae) and evaluated their antioxidant potential, anti-tyrosinase and antiproliferative activities in vitro and its anti-inflammatory function in vivo^26^. However, the antimicrobial effects of extracts containing sterols from edible insects is unknown. This study aimed to identify and compare sterol composition from selected edible insects and screen for their antimicrobial properties.

## Results

### Identification and classification of sterols from edible insects

The GC–MS data for the DCM extracts of the investigated edible insects represented mainly sterols and fatty acids. With reference to the retention times, fatty acids were found in the range between 20 and 30 min, whereas sterols appeared between 34 and 42 min. To attain the study’s aim of identifying sterols, 19 different sterols/stanols were classified from the extracts. They all featured 27–30 carbon atoms with 0–3 double bonds. The identification of these sterols was done based on GC–MS library data, molecular ion (*m/z*) values and literature reports.

Cholesterol was found to be the most prevalent sterol in all of the insect samples, except for *H. illucens*. The three most common phytosterols in the insect extracts (with the exception of *B. mori* and *Macrotermes* sp.) were *γ*-sitosterol, campesterol, lathosterol and stigmasterol. They are classified as either methylsterols or ethylsterols depending on the type of group attached at the (C-24) carbon atom side chain. Stigmasterol was mainly found in *H. illucens, S. gregaria, and P. sinuata.* Lathosterol, on the other hand, was found in both the crickets (*G. bimaculatus* and* S. icipe*), *P. sinuata* and *S. gregaria* extracts, whereas desmosterol was only found in *R. differens* and *G. krucki* extracts. The notable difference in sterol diversity between the two cricket extracts was the presence of taraxasterol in *G*. *bimaculatus* (Table 1)*.* From the mass spectrometry data, its molecular ion (M^+^, *m/z* 426.4) corresponded to 30 carbon atom with 1 double bond (30:1, Table 1).

**Table 1:** Diversity of sterols (phytosterols/stanols) in the insect extracts.

	RT (min)	Sterol type	Cn:DB	Δ^DB^	Samples detected	M^+^
1	34.36	27-*Nor*-ergosta-5,22-dien-3-ol(3*β*, 22*Z*)	27:2	Δ^5,22^	*G. bimaculatus, S. icipe, Macrotermes *sp.	384.3
2	34.93	Cholesterol	27:1	Δ^5^	All (100%)	386.4
3	35.09	Cholestanol	27:0	0	*P. sinuata*	388.4
4	35.62	Desmosterol	27:2	Δ^5,24^	*G. krucki*, *R. differens*	384.3
5	35.73	Ergosta-5,22-dien-3-ol (3*β*, 22*E,* 22*S*)	28:2	Δ^5,22^	*Macrotermes *sp.	398.3
6	35.84	Lathosterol	27:1	Δ^7^	*G. bimaculatus, S. icipe, S. gregaria, P. sinuata*	386.4
7	36.75	Ergost-22-en-3-ol (3*α*, 5*β*, 22*E*)	28:1	Δ^22^	*P. sinuata*	400.4
8	36.83	Campesterol	28:1	Δ^5^	*G. bimaculatus, S. icipe, S. gregaria, P. sinuata, R. differens*	400.4
9	36.88	*γ*-Ergostenol (Tr)	28:3	Δ^5,7,22^	*H. illucens*	396.4
10	37.05	Ergostanol	28:0	0	*P. sinuata*	402.4
11	37.29	Cholest-4-en-3-one	27:2	Δ^3,4^	*S. icipe, B. mori*	384.3
12	37.31	Cholesta-3,5-diene	27:2	Δ^3,5^	*G. bimaculatus*	368.3
13	37.52	Stigmasterol	29:2	Δ^5,22^	*H. illucens, S. gregaria, P. sinuate*	412.4
14	38.79	*γ*-Sitosterol	29:1	Δ^5^	*G. bimaculatus, S. icipe, P. sinuata, G. krucki*	414.4
15	38.80	*β*-Sitosterol	29:1	Δ^5^	*S. gregaria*	414.4
16	38.81	Stigma-7-en-3-ol (3*β,* 5*α*, 24*S*)	29:1	Δ^7^	*H. illucens, R. differens*	414.4
17	39.03	Stigmastanol	29:0	0	*P. sinuata*	416.4
18	39.21	24-Propylidenecholest-5-en-3*β*-ol	30:2	Δ^5,24^	*H. illucens*	426.4
19	40.64	Taraxasterol (Tr)	30:1	Δ^20^	*G. bimaculatus*	426.4

Tr indicates < 70% quality, *RT (min)* retention time (minutes), *Cn:DB* carbon number:double bonds, *Δ DB* double bond position, *M*^*+*^ molecular ion peak. The reference library used is NIST11.

**Figure 1:**
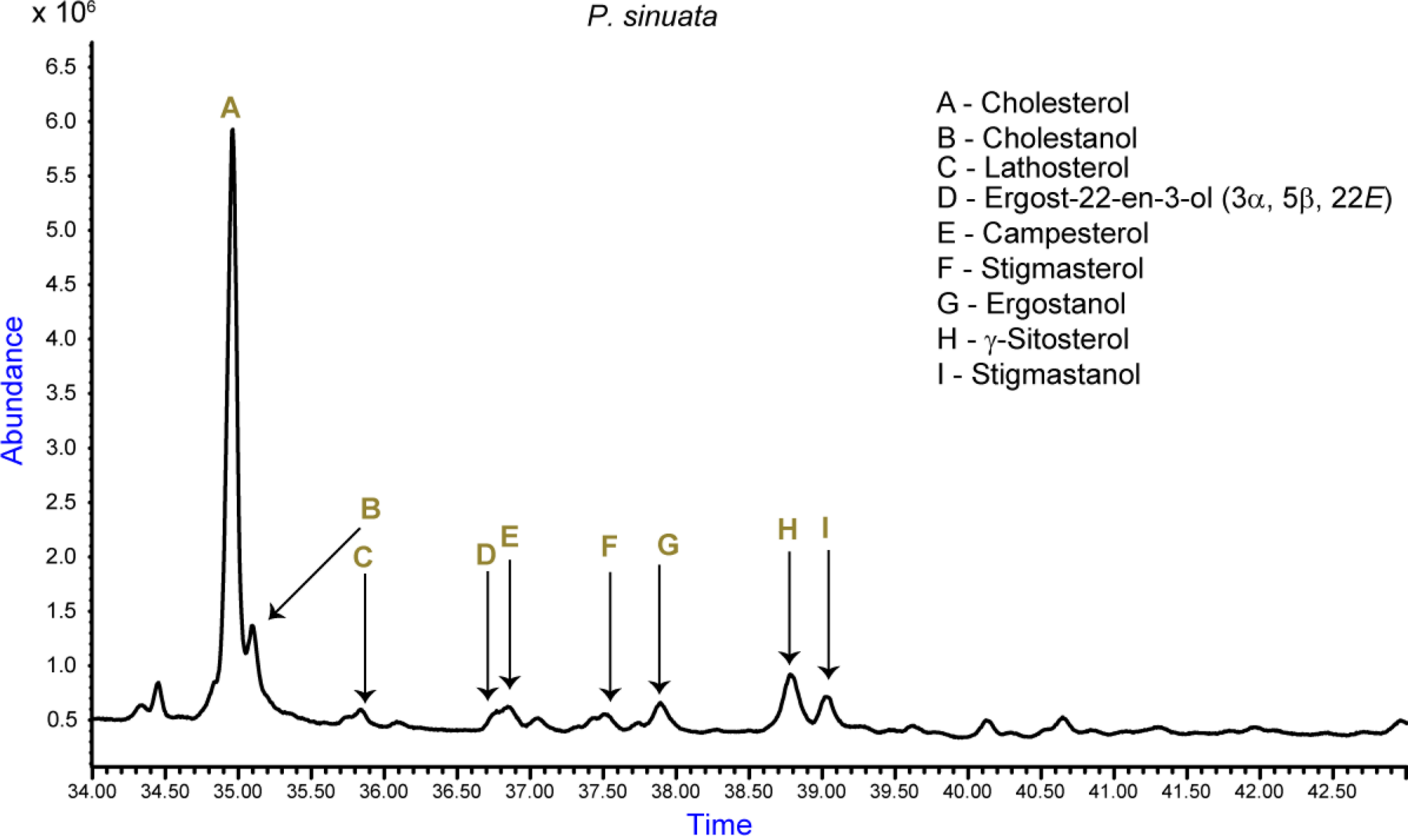
Total ion chromatogram indicating the abundance of phytosterols/stanols identified in African fruit beetle larvae *P. sinuata*.

The identified stanols (saturated form of sterols) were cholestanol (27:0, M^+^, *m/z* = 388.4), ergostanol (28:0, M^+^, *m/z* = 402.4) and stigmastanol (29:0, M^+^, *m/z* = 416.4), all of which were discovered from the *P. sinuata* extract (Table 1; Fig. 1).

**Figure 2:**
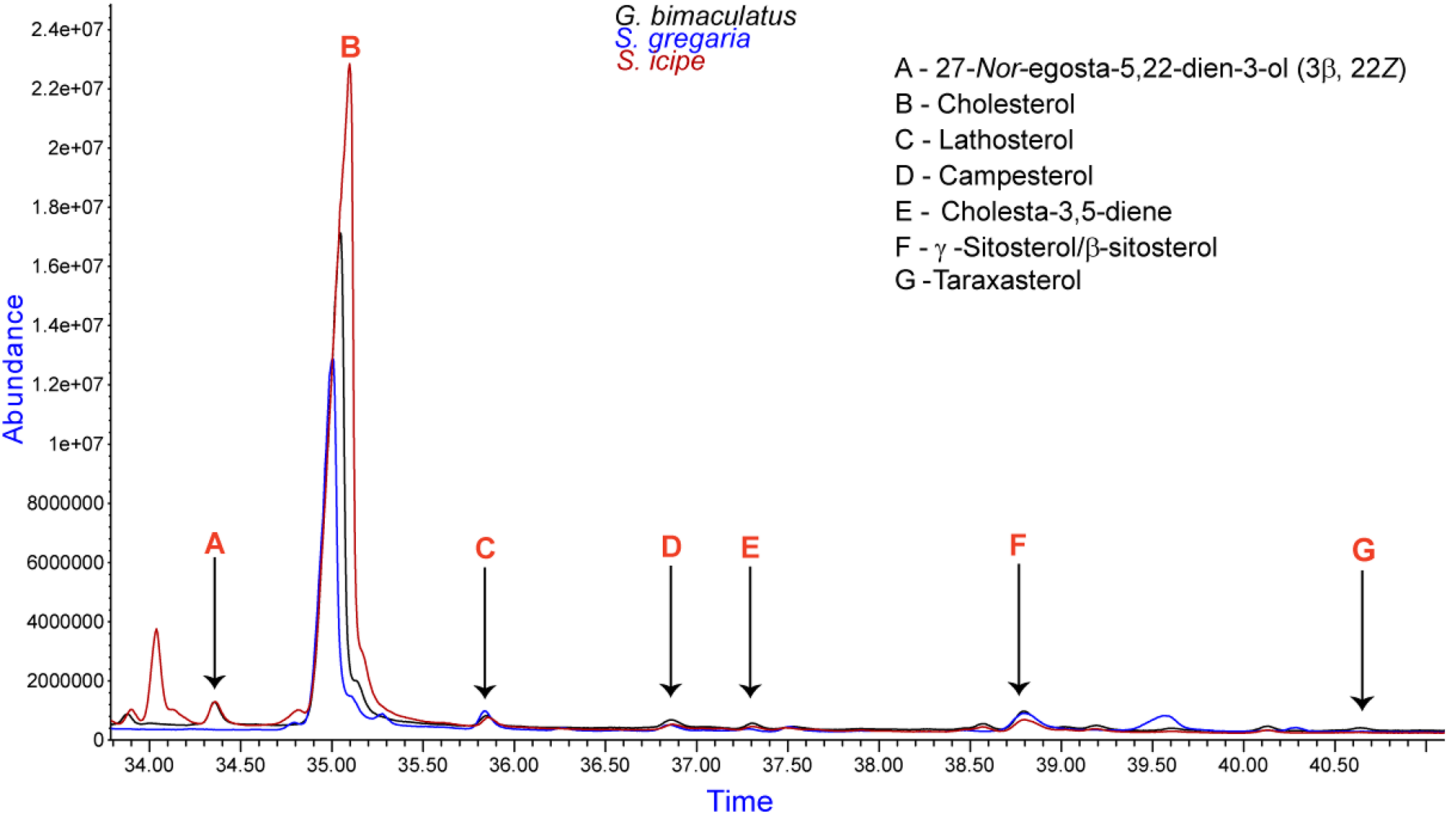
Abundance of sterols in crickets: *G. bimaculatus, S. icipe* and the desert locust *S. gregaria*.

**Figure 3:**
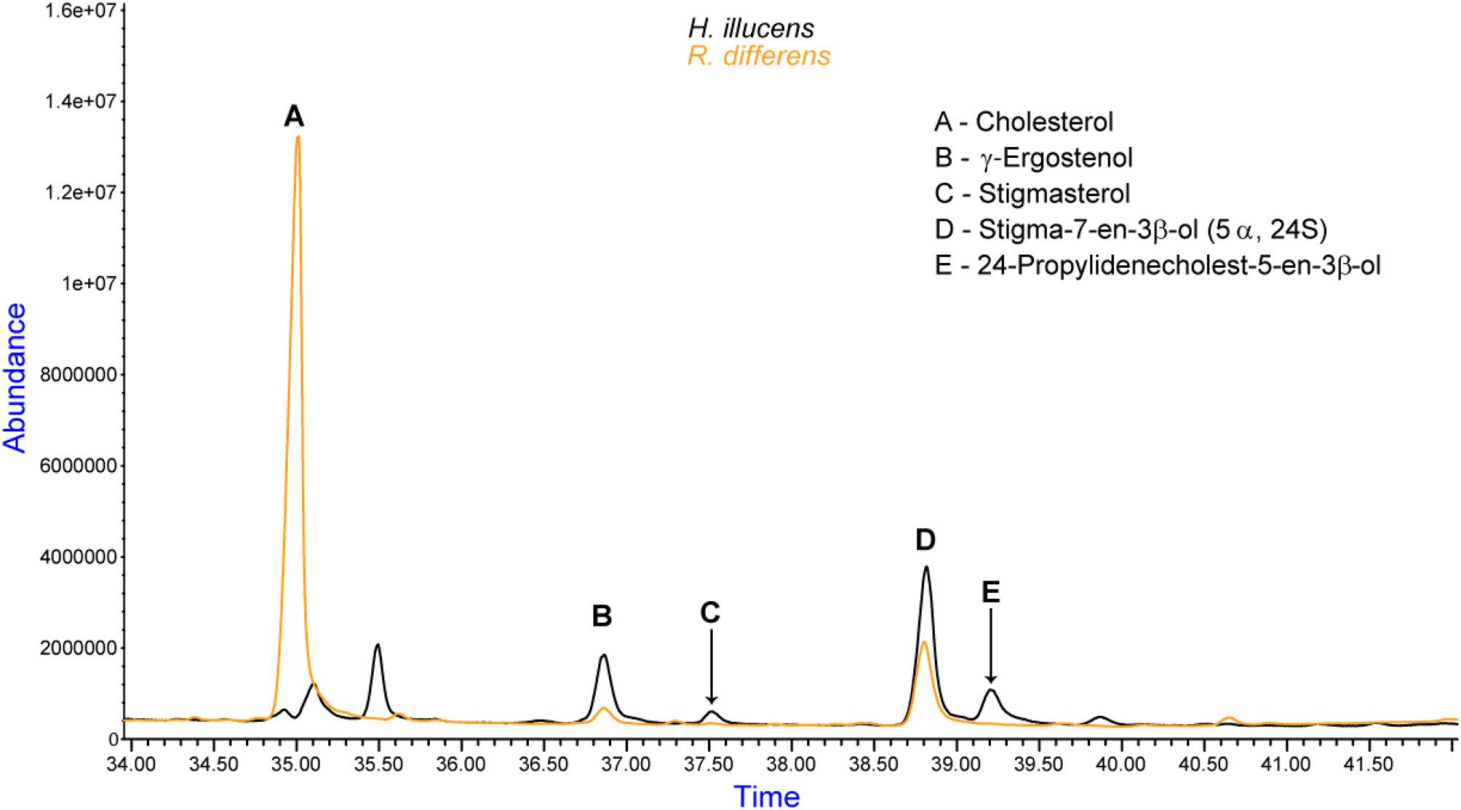
Quantification of the stigma-7-en-3*β*-ol sterol in *H. illucens* and *R. differens*.

Based on the abundance of different sterols in the samples, cholesterol’s highest value was at 2.3 × 10^7^ as noted in the *S. icipe* sample compared to the closely related species *G. bimaculatus* at 1.7 × 10^7^ absorption units (Fig. 2). As well, stigma-7-en-3*β*-ol (*5α*, *24S*), was mainly identified in the *H. illucens* and the *R. differens* extracts. Its abundance was recorded to be 4.0 × 10^6^ and 2.0 × 10^6^ absorbance units, respectively (Fig. 3). Further analysis revealed that the peak at 36.86 min contained two sterols, that is, campesterol (quality > 70%) at 36.88 min and traces of γ-ergostenol at 36.83 min (quality < 70%).

A comparative analysis showed that, the two cricket *G. bimaculatus* and *S. icipe* have cholesta-3,5-diene and cholest-4-en-3-one at 37.31/37.29 min, respectively (Fig. 2)*.* Additionally, the phytosterol profile portrayed by *S. gregaria* was similar to that of the crickets with the exception that *β*-sitosterol replaces *γ-*sitosterol and the absence of 27-*Nor*-ergosta-5, 22-dien-3-ol (*3β, 22Z*) (Fig. 2).

**Figure 4:**
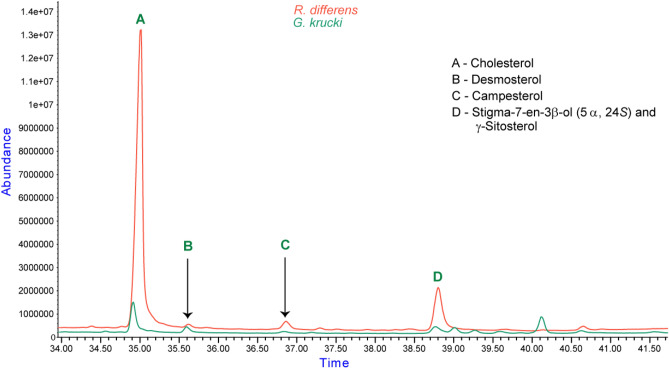
An overlay of *R. differens* and *G. krucki* showing the presence of desmosterol (labelled B). The peak denoted by letter D contained different sterols at 38.79 (*γ*-sitosterol) and at 38.81 (stigma-7-en-3*β*-ol (5*α*, 24S) in *G. krucki* and *R. differens.*

**Figure 5:**
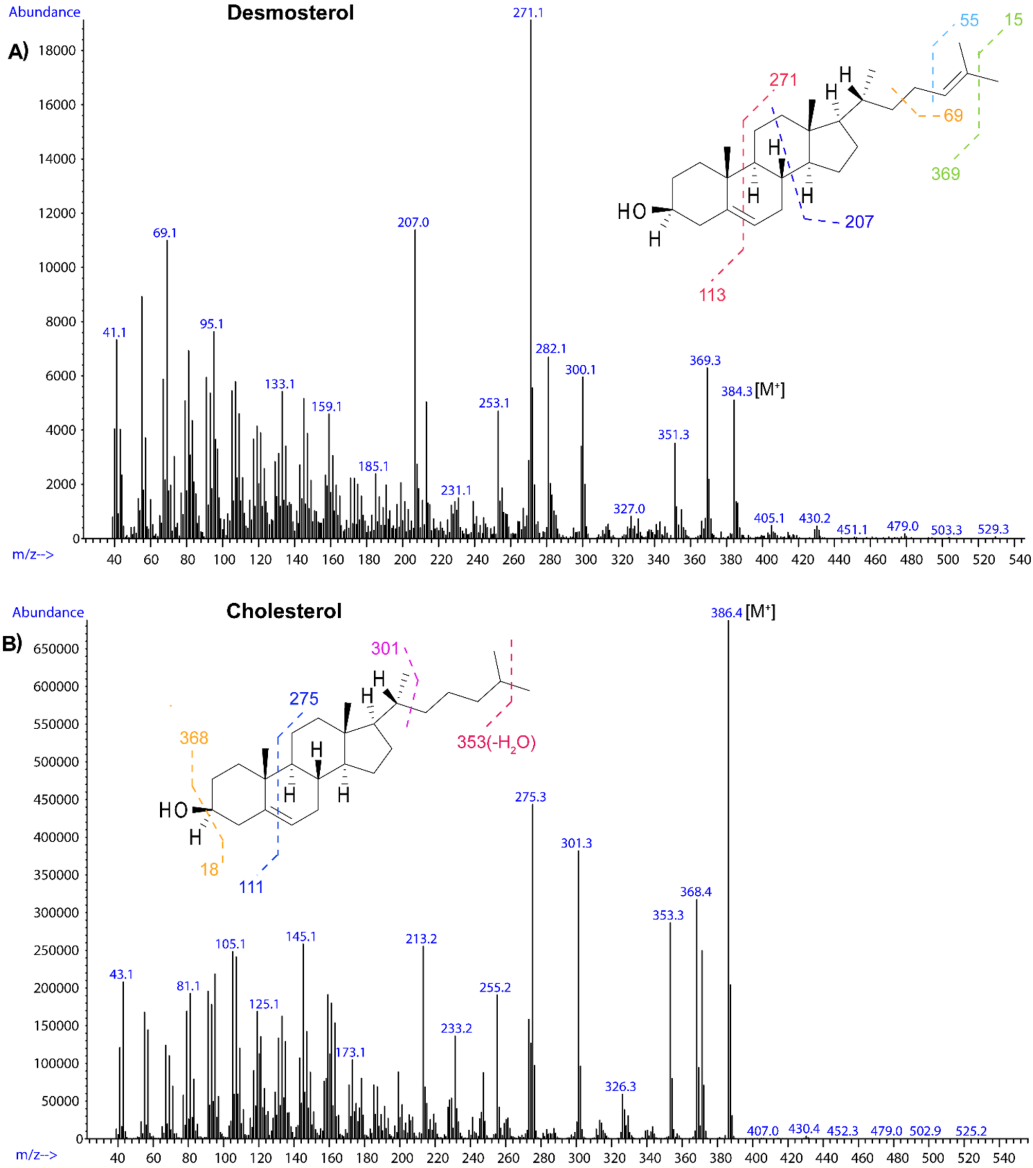
Mass spectra of (**A**) desmosterol and (**B**) cholesterol indicating the possible fragment peaks.

The extracts of *R. differens and G. krucki* commonly contained desmosterol (Fig. 4). Desmosterol (Δ^5,^^24^) is an intermediate product in the biosynthesis of cholesterol with characteristic molecular ion peaks of *m/z* 253, 271, 300 and 384. The mass spectrum showed largely analogous fragmentation, with the mass spectrum for desmosterol containing a distinctive *m/z* 271 ion for the loss of 113 Da (C_8_H_15_), which would indicate possibility of an unsaturated sterol side chain. The other molecular ion peaks corresponded to the shown fragments 253 [M^+^−C_8_H_18_O], 300 [M^+^−C_6_H_12_], 369 [M^+^−CH_3_] (Fig. 5A). The fragmentation pattern was compared to that of cholesterol as shown in Fig. 5B providing evidence for the unsaturation in desmosterol. Cholesterol portrayed molecular ion peaks at m/z 368.4 [M^+^−CH_3_], 353.3[M^+^–(CH_3_ + H_2_O)] and 275(C_20_H_35_^2·+^) showing a loss of 113 Da.

**Figure 6:**
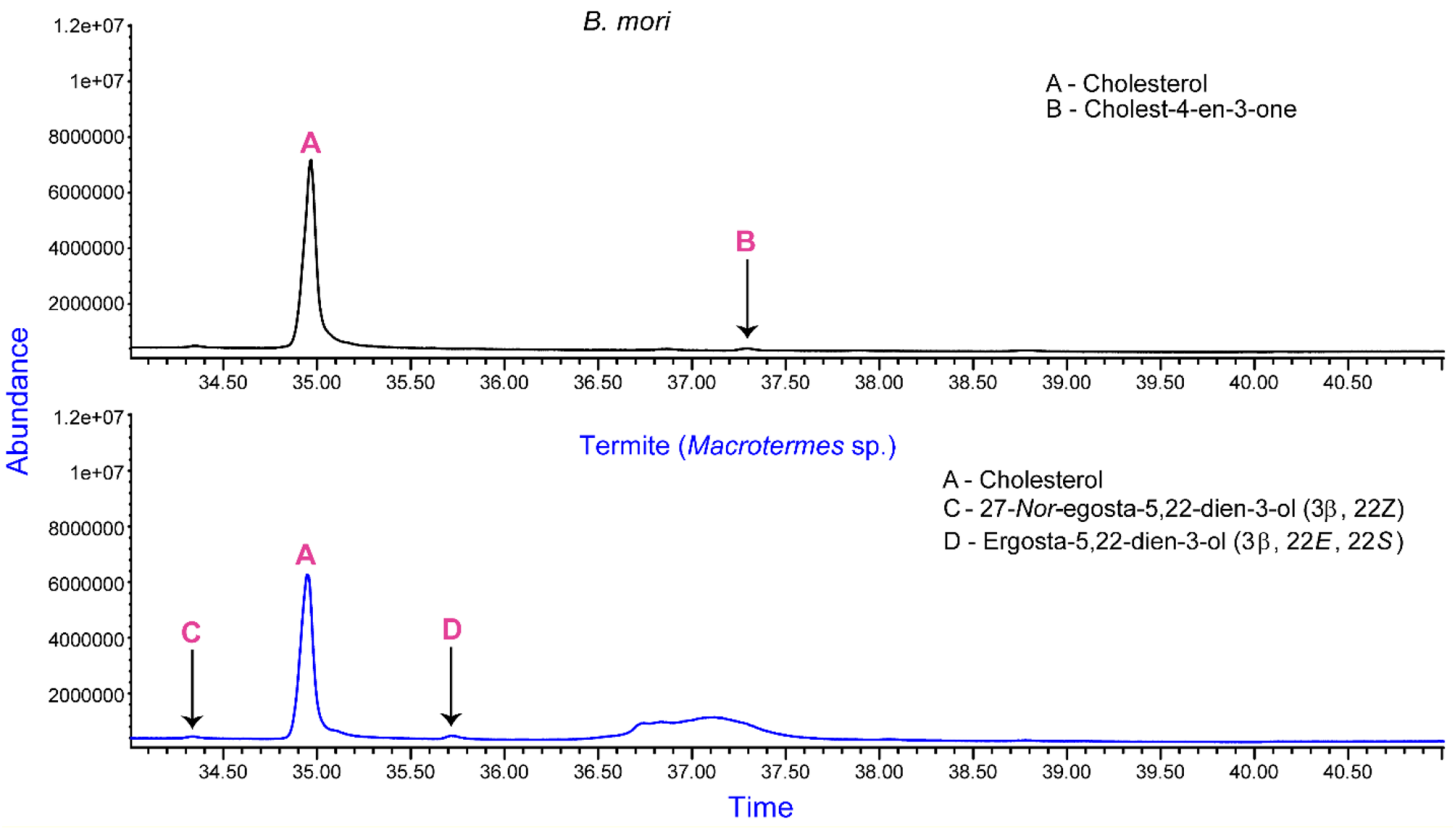
Chromatogram indicating sterols from silkworm-*B. mori* and *Macrotermes *sp*.* extracts.

Although there is paucity of information regarding the origin of phytosterols in invertebrates, certain insects are exceptional since they are known to acquire them from their dietary sources as shown by desert locust^27^. However, *B. mori* larvae, which feeds mainly on mulberry leaves (*Molus alba*), only portrayed cholesterol and cholest-4-en-3-one as the present sterols (Fig. 6).

**Figure 7:**
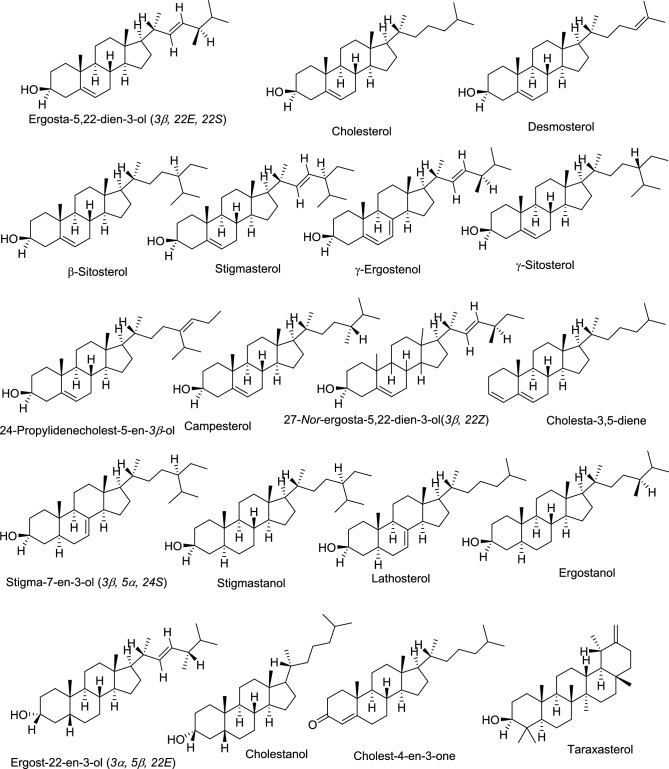
The chemical structures of the identified sterols in the selected edible insects.

Cholesterol was the predominant sterol found in *Macrotermes* sp., with 27-*Nor*-ergosta-5,22-dien-3-ol(3*β*, 22*Z*) and ergosta-5,22-dien-3-ol (*3β*,* 22E*,* 22S*) as minor peaks (Fig. 6). It is possible that their diet dictated the lack of additional phytosterols or most of them were converted to cholesterol. All the identified sterols have been summarized in Fig. 7 below.

### Antimicrobial effects of the sterol containing extracts

Antimicrobial activity tests were performed on the DCM fraction of the different insect extracts. The comparison of the inhibition zones of the extracts to the positive control revealed that they exhibited varying inhibitory zones. When the DCM dissolved samples were exposed to *B. subtilis*, *S. icipe* exhibited an appreciable inhibitory zone of 8.33 ± 0.58 mm as the highest amongst all the extracts of the extract from *S. gregaria* portrayed moderate inhibition zones (7.0 ± 0.58 mm) whereas *R. differens* and *G. Krucki* had the least inhibitory effects (6.33 ± 0.58 mm) against *B. subtiilis*. When the cricket extracts were subjected to *E. coli*, the inhibition zone of *G. bimaculatus* was determined to be 6.67 ± 0.58 mm whereas *S. icipe* exhibited no appreciable activity*.* The *H. illucens* extract (containing 98.4% fatty acid content) was the most active against *E. coli* exhibiting 8.0 ± 1.00 mm as the inhibition diameter. On the contrary, *B. mori* extract with 98.2% fatty acid content exhibited moderate inhibitory diameter against all the test pathogens.

A comparison of the antibacterial activity was carried out by dissolving the sample in 20% of acetonitrile and subjecting to the test organisms. Markedly, *S. gregaria* had a 8.67 ± 0.58 mm inhibition zone against *B. subtilis*, while *H. illucens* the second highest had 7.67 ± 0.58 mm inhibition when exposed to *B. subtilis* pathogen. The exposure of *E. coli* to the extracts proved that P. sinuata was the most active with 7.67 ± 0.58 mm inhibitory effects. The extracts of *R. differens* and *B. mori* however, did not exhibit any significant antibacterial activity against *E.coli*. On the other hand, *S. icipe*, was the least active extract, with no discernible inhibition zone against *B. subtilis* and *S. aureus* organisms. It however indicated a 7.0 ± 1.00 mm inhibitory activity against *E. coli* (Table 2). Thus, the results appear to be consistent regardless of the solvent used in dissolving the extracts.

**Table 2:** Antimicrobial activities of the extracts containing the different sterols against three test organisms.

Code	Sample\test organism	Conc. (mg/disc)	Inhibition zones (diameter, mm)					
			*B. subtilis*		*Methicillin-susceptible S. aureus 25923*		*E. coli* 25922	
			A	B	A	B	A	B
1	Long-horned grasshoppers (*R. differens*)	0.2	6.33 ± 0.58^ab^	6.33 ± 0.58^b^	7.67 ± 0.58^ad^	0 ± 0.00^a^	7.0 ± 1.00^bc^	0 ± 0.00^a^
2	African fruit beetle (*P. sinuata*)	0.2	7.33 ± 0.58^ac^	6.67 ± 0.58^b^	6.33 ± 0.58^ab^	7.0 ± 0.00^b^	6.33 ± 0.58^bc^	7.67 ± 0.58^b^
3	Cricket (*S. icipe*)	0.2	8.33 ± 0.58^c^	0 ± 0.00^a^	6.67 ± 0.58^ac^	0 ± 0.00^a^	0 ± 0.00^a^	7.0 ± 1.00^b^
4	Caterpillar (*G. krucki*)	0.2	6.33 ± 0.58^ab^	6.33 ± 0.58^b^	6.67 ± 0.58^ac^	7.0 ± 0.00^b^	7.67 ± 0.58^bc^	6.33 ± 0.58^b^
5	Termite (*Macrotermes *sp.)	0.2	7.67 ± 0.58^bc^	7.33 ± 0.58^bc^	7.0 ± 1.00^ad^	6.67 ± 0.58^b^	7.67 ± 0.58^bc^	7.33 ± 0.58^b^
6	Black Soldier Fly (*H. illucens*)	0.2	8.0 ± 0.00^c^	7.67 ± 0.58^bc^	8.67 ± 0.58^b^	0 ± 0.00^a^	8.0 ± 1.00^c^	7.33 ± 0.58^b^
7	Locust (*S. gregaria*)	0.2	7.0 ± 0.00^ac^	8.67 ± 0.58^c^	8.0 ± 1.00^bcd^	7.0 ± 0.00^b^	8.0 ± 1.00^c^	6.67 ± 0.58^b^
8	Cricket (*G. bimaculatus*)	0.2	7.33 ± 0.58^ac^	6.67 ± 0.58^b^	8.33 ± 0.58^cd^	6.33 ± 0.58^b^	6.67 ± 0.58^bc^	6.67 ± 0.58^b^
9	Silkworm (*B. mori*)	0.2	7.67 ± 0.58^bc^	6.33 ± 0.58^b^	7.33 ± 0.58^ad^	6.33 ± 0.58^b^	6.67 ± 0.58^bc^	0 ± 0.00^a^
Pos:	Oxy*	0.02	21.0 ± 1.00^d^	19.67 ± 1.16^d^	8.33 ± 0.58^cd^	7.0 ± 0.00^b^	20.33 ± 0.58^d^	25.0 ± 1.00^c^
Neg:	A. DCM	–	6.0 ± 0.00^a^	–	6 ± 0.00a	–	6 ± 0.00b	–
	B. 20% ACN	–	–	n.i	–	n.i	–	n.i
F value			152.8	217.1	5.729	386.1	149.5	389.0
p value			< 2 × 10^–16^	< 2 × 10^–16^	3.18 × 10^–4^	< 2 × 10^–16^	< 2 × 10^–16^	< 2 × 10^–16^

Mean values of triplicate inhibition zones and their standard deviations. Values with the same superscript in the same column are not significantly different at p < 0.05 and d.f (10, 22). *Positive controls used (Oxy = Oxytetracycline), Negative controls (*DCM* dichloromethane, *20% ACN* acetonitrile supplemented by 0.1% DCM), *n.i* no inhibition samples using 20 µL of the given concentration (10 mg/mL) solution. The inhibition values for positive controls were obtained with 20 µL of 1 mg/mL solution. All the values are means of triplicate experiments and their standard deviations.

From the MBC results, it is evident that *S. icipe* extract proved to be the most active followed by *G. bimaculatus. Macrotermes *sp. showed moderate inhibition concentration (2.5 mg/mL) and *R. differens* showed the least activity against all the test organisms. Analysis of the activity across individual test organisms reveal that *G. bimaculatus* was more potent against *S. aureus*, *S. icipe* and *G. Krucki* against *E.coli*. The most potent extract against *B. subtilis* was found to be *S. icipe*. All the extracts exhibited no growth at a concentration of < 0.312 mg/mL (Table 3). Indeed, in general lower MBC values were recorded against *E. coli* (< 0.312–2.5 mg/mL). This indicates that *E. coli* was more susceptible to the active components in the extracts than the other test organisms. This study provides an insight into the value of insects and their chemical components such as lipids.

**Table 3:** Minimum bactericidal concentration observed after serial dilution of the extracts.

Test organism	MBC (mg/mL)								
	1	2	3	4	5	6	7	8	9
*S. aureus*	5.0	2.5	0.63	1.25	2.5	5.0	2.5	< 0.31	0.63
*E. coli*	2.5	0.63	< 0.31	< 0.31	2.5	1.25	0.63	0.63	1.25
*B. subtilis*	2.5	5.0	< 0.31	2.5	2.5	2.5	2.5	2.5	5.0

*MIC ≡ MBC* in (mg/mL) for extracts (**1**–**9**) using 40 μL of 10 mg/mL solution and 40 μL MHB medium, respectively. Streptomycin as the positive control at 1 mg/mL (40 μL) with no indication of growth at all serial concentrations used. No growth was observed in the negative control (5% DMSO).

## Discussion

The evaluation of sterol richness and composition in edible insects revealed cholesterol to be the most abundant in majority of the samples. This could be attributed to its lipophilic nature and significance in the structural makeup of the cell membrane in living organisms thus modulating fluidity^24^. On the contrary, its reduced quantity in *H. illucens* could be due to the elevated fatty acid content, which might have obscured its biosynthesis since they have a common starter building block unit i.e. acetyl -CoA. The presence of stanols in *P. sinuata* is attributed to the action of the hydrogenase enzyme in the insect’s body/gut. The minor structural variations between sterols and stanols may have a distinct impact on their functions and metabolisms. Moreso, their biotransformation could be related to the differences of individual phenotypes and the composition of gut microbiota present in the insects^28^.

The higher quantities of campesterol/*γ*-ergostenol, stigma-7-en-3*β*-ol (*5α, 24S*), and 24-propylidenecholest-5-en-3*β*-ol in the *H. illucens* extract could be linked to the presence and the action of oxidoreductases on the sterol side chain^29^. Among these sterols, Giner et al.^29^ found out that 24-propylidenecholest-5-en-3*β*-ol was produced in about 17 species of marine algae as a novel sterol. These findings are supported by the work by Vidal et al.^30^ where oxidoreductases are named as key and most abundant enzymes in the catalysis of approximately one-third enzymatic activities found in BRaunschweig Enzyme Database (BRENDA).

Cholest-4-en-3-one and cholesta-3,5-diene metabolites found in *G. bimaculatus, S. icipe* and *B. mori* are known to be transformed products from cholesterol. In particular, cholest-4-en-3-one is an intermediate product of the transformation process of cholesterol to coprostanol under anox conditions via oxygenase-independent reactions as established in bacteria^31^. On the contrary, cholesta-3,5-diene is a sole primary product when cholesterol is subjected to high temperatures > 300 °C.

The analysis of *B. mori* extract showed an incomplete profile from that depicted from mulberry leaves. The mulberry leaves have been documented to possess the following phytosterols: cholesterol, stigmasterol, sitosterol and campesterol^32^. The identification of the two sterols from *B. mori* as shown by this study could suggest that the dietary sterols in mulberry may have been converted into cholesterol and cholest-4-en-3-one depending on the larval stage investigated^33^. Thus, the molecular conversion of phytosterols and the metabolism of *B. mori* larvae remains to be fully elucidated and understood.

Furthermore, termites primarily depend on wood to obtain cellulose and nutrients that they need for survival. However, it is noted that sterol composition is crucial for cellulose biosynthesis as it is linked to cell wall formation^34^. As a result, more research should be directed towards determining all the sterols generated from cholesterol modifications employing various sterolomic approaches^35^.

**Figure 8:**
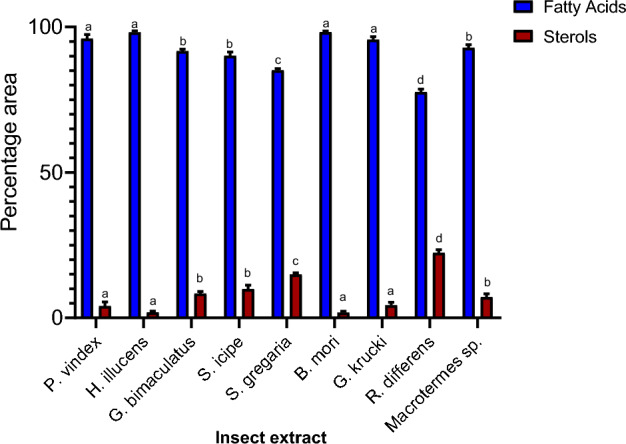
The percentage area mean (± SE) of fatty acids and sterols in the DCM extracts. Bars are capped with different letters shows how significantly the given data correlate (Tukey's HSD test: p < 0.05).

This study showed an elevated fatty acid content (98.4%) in *H. illucens* extract (Fig. 8) which may be attributed to the large inhibitory zone observed. According to literature, the fatty acid content of *H. illucens* is estimated to be 30% most of which are categorized as antimicrobial lipids^36,37^. However, the *B. mori* extract with 98.2% of the fatty acid content exhibited moderate activity. It is imperative that further studies be carried out to ascertain the effect of fatty acid in combination with the specific sterols identified. The study also points out that stigmasterol is the common sterol in *H. illucens* and *S. gregaria* both of which have the highest activity against *MSSA* 25923 and *E. coli*. In a study by Gade et al.^38^, stigmasterol was found to be the main compound responsible for the observed larvicidal activity against *C. quinquefasciatus* and *A. aegypti*.

Moreover, the high activity observed in *S. icipe* and *G. bimaculatus* could be attributed to the presence of 27-*Nor*-ergosta-5,22-dien-3-ol(3*β*, 22*Z*). Previous studies have shown that ergosterol derivatives have the potential to exhibit antibacterial, antitumor, cytotoxic, rheumatoid arthritis and even immune promoting properties^39^. For instance, when ergosterol and cholesterol were combined with aminoglycosides and tested against multi-resistant bacterial strains, the activity of the aminoglycoside increased with higher sub-inhibitory concentrations of the sterols^40^.

Additionally, taraxasterol that was present in *G. bimaculatus* has been reported in literature to possess many important pharmacological actions that include anti-cancer, anti-allergic, anti-oxidant, and anti-inflammatory activities^41–44^. Therefore, it may be responsible for the enhanced antibacterial activities of *G. bimaculatus* (9 mm) against *MSSA 25923* in comparison to *S. icipe* (6 mm). These results are in line with a study from which twelve triterpenoid substances, including taraxasterol, were isolated and purified from Mexican Asteraceae plants. Only taraxasterol molecule was found to have antibacterial activity against *S. aureus*^45^.

It is therefore important to understand the plausible biosynthesis of the identified sterols. There are three key phases in the production of (C-30) sterols starting from squalene as delineated in literature^5^. The first stage entails six steps that include:

1. The conversion of acetyl CoA to acetoacetyl CoA mediated by the enzyme acetoacetyl CoA thiolase (AACT).
2. Acetoacetyl CoA is converted into 3-hydroxyl-3-methylglutaryl CoA catalyzed by hydroxyl-3-methylglutaryl CoA synthase (HMGS).
3. 3-Hydroxy-3-methylglutaryl CoA reductase (HMGR) converts 3-hydroxy-3-methylglutaryl CoA into mevalonic acid (MVA).
4. Phosphomevalonate kinase (PMK) converts mevalonic acid (MVA) into phosphomevalonate.
5. Phosphomevalonate kinase (PMK) converts phosphomevalonate into diphosphomevalonate.
6. Mevalonate diphosphate decarboxylase (MVD) then converts diphosphomevalonate to isopentyl diphosphate (Δ^3^-IPP).

The two-phosphorylation events at MVA's C-5 and a decarboxylation/elimination step changes MVA into IPP in the first stage; IPP, the basic C-5 building block, that is then added to the prenyl diphosphate co-substrates to generate longer chains.

**Figure 9:**
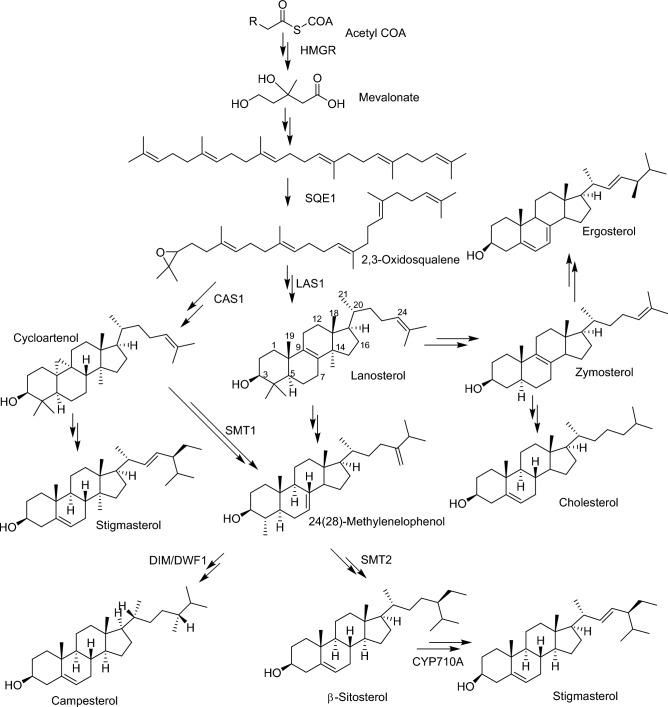
Biosynthesis of selected sterols identified from the edible insects. *HMGR* 3-hydroxy-3-methylglutaryl-CoA reductase, *SQE1* squalene epoxidase, *CAS1* cycloartenol synthase, *LAS1* lanosterol synthase, *SMT1/2* C24-sterol methyltransferase, *DIM/DWF1* sterol-∆^24^-isomerase/reductase, *CYP710A* C-22-sterol desaturase. The double arrows indicate several steps of enzymatic reactions.

The condensation reaction is repeated in the second stage with the addition of Δ^3^-IPP, yielding the C-15 allylic product farnesyl diphosphate. By the action of squalene synthase (SQS), two molecules of farnesyl diphosphate condense tail to tail into the C-30 acyclic polyene squalene. A NADPH-dependent mono-oxygenase reaction catalyzed by squalene epoxidase (SQE) converts the C-30 symmetric olefin to *S*-oxidosqualene, which is then cyclized by an oxidosqualene sterol synthase to generate the steroidal backbone structure as represented in lanosterol (Fig. 9). Lanosterol is transformed to cholesterol in the third stage. Conversely, the cycloartenol synthase (CAS) pathway is thought to be mostly a plant sterol pathway converting oxidosqualene to cycloartenol^14^. The enzymatic activities of sterol methyltransferases (SMT), which catalyze the methylation reactions at the (C-24) carbon atom in the side chain, are used to elucidate the mechanisms of variations in the ratio of molecular kinds of sterols such as campesterol and *β*-sitosterol^46^ (Fig. 9).

Alternatively, it is postulated that cholesterol in insects can be synthetized via the enzymatic conversion pathway from *β*-Sitosterol. Here, *β*-sitosterol is first converted to fucosterol then to 24,28-epoxyfucosterol and desmosterol as intermediates. With the action of 24-reducing enzyme on desmosterol as a rate-limiting step, cholesterol is formed^33^.

**Figure 10:**
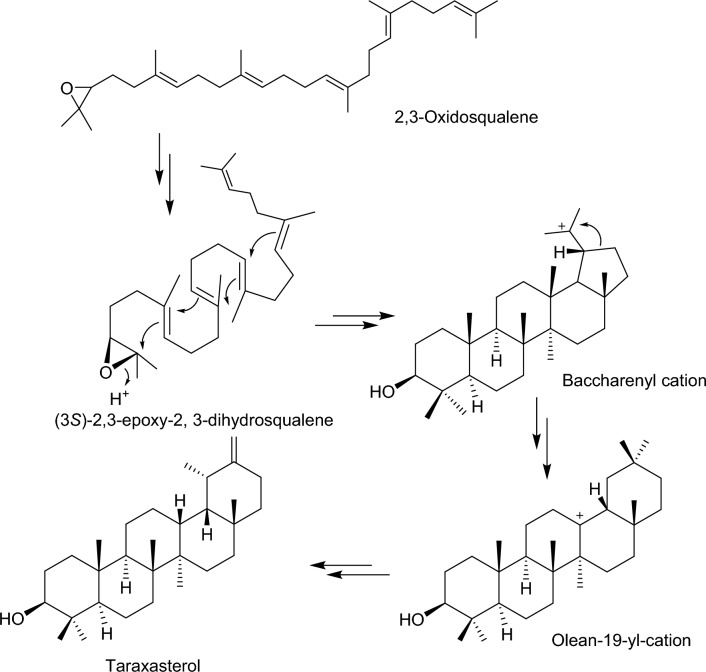
Proposed biosynthesis of taraxasterol identified from the cricket species—*G. bimaculatus.*

Taraxasterol on the other hand originates from squalene to (3*S*)-2,3-epoxy-2,3-dihydrosqualene mediated by oxidosqualene cyclases enzymes. The (3*S*)-2,3-epoxy-2,3-dihydrosqualene is then converted to olean-13-yl cation, which undergoes a series of rearrangement reactions to give taraxasterol (3*β*; 18*α*; 19*α*;)-Urs-20(30)-en-3-ol)^44^ (Fig. 10).

In conclusion, we herein describe the first comparative study of sterols in edible insect extracts and indicate their potential antibacterial effects. The range of sterols identified in the various extracts were between 2 and 9 different types. Cholesterol was the most abundant sterol in all the extracts except in *H. illucens*. Extract obtained from *P. sinuata* portrayed an array of phytosterols as well as stanols. The sterol 24-propylidenecholest-5-en-3*β*-ol, which has been widely identified in green algal species, was only found in *H. illucens* extract. On the other hand, taraxasterol (known to possess anti-cancer, anti-allergic, anti-oxidant, and anti-inflammatory activities) was identified in *G. bimaculatus* extract. The extracts from the evaluated insects showed significant inhibitory activities against two clinically important (Methicillin-susceptible *S. aureus* 25923, *E. coli* 25922) and one indicator (*B. subtilis*) pathogens. On this background, products containing sterols from edible insects could be utilized as targets for drug discovery against disease causing pathogens. It is therefore recommended that further studies on the isolation of individual sterols from the insects be carried out to investigate their antibacterial effects. The potent phytosterols could be used to formulate products that can help in improving health status of people living in low and middle-income countries. Moreover, structure activity relationship (SAR) studies could be carried out on the different bioactive phytosterols (in line with the biosynthetic pathway) to improve the observed activity. Lastly, varying the rearing or diet conditions of these insects is suggested to improve their mass production and increase biodiversity of the sterols as a sustainable source.

## Materials and methods

### Materials

All the solvents used in the study that include LC–MS grade methanol (MeOH), water (H_2_O), HPLC grade dichloromethane (DCM) and hexane were purchased from Merck (Darmstadt, Germany).

### Insect rearing

The insects, black soldier fly (*H. illucens),* cricket (*G. bimaculatus and S. icipe*), desert locust (*S. gregaria*), silkworm (*B. mori*), African fruit beetle (*P. sinuata*), caterpillar (*G. krucki*), long-horned grasshopper (*R. differens*) and termite (*Macrotermes* sp.) used in the experiment were reared in the Insect and Animal Rearing and Quarantine unit at the International Centre of Insect Physiology and Ecology (*icipe,* 01° 13′ 25.3″ S, 36° 53′ 49.2″ E; ≈ 1600 m ASL), except termites, which were sourced from the wild at Kakamega County, Kenya. The institution (*icipe*) has a designated insectary unit where mass rearing of insects is done and they are fed on locally cultivated plants some of which are common cash crops in Kenya. Specifically, these insects were fed on various diets: *H. illucens* on Potato waste, cricket (*G. bimaculatus and S. icipe)* on cassava leaves, *S. gregaria* on wheat bran, *B. mori* on mulberry leaves, *P. sinuata* on cattle manure, *G. krucki* on mango leaves*,* and *R. differens* on panicum grass.

### Insect extract preparation

Before commencement of the experiment, each insect sample was properly cleaned to remove the debris. The samples were then placed in an oven at 60 °C for at least 48 h. The dried insects were ground to obtain fine powder using a blender. Approximately 10 g of each ground sample were extracted with 80% methanol and evaporated in vacuo. To the residual aqueous phase, about 50 mL of distilled water was added and partitioned with equal volume of *n*-hexane to remove the fatty acids. This was followed by subsequent extraction using equal volume of DCM. The DCM soluble extract was concentrated in vacuo and the sample prepared for GC–MS analysis by making a concentration of 100 ng/μL in triplicates.

### GC–MS instrument conditions

Samples were analyzed by GC on a 7890A gas chromatograph (Agilent Technologies, Inc., Santa Clara, CA, USA) coupled to a 5975C mass selective detector (Agilent Technologies, Inc., Santa Clara, CA, USA). The analysis was done using the following conditions: inlet temperature was set at 270 °C, transfer line temperature at 280 °C, and column oven temperature was programmed from 35 to 285 °C with the initial temperature maintained for 5 min then 10 °C/min to 280 °C for 10.5 min. The final temperature was set at 50 °C/min to 285 °C and held at this level for 29.9 min. The GC was fitted with a HP-5 MS low bleed capillary column (30 m × 0.25 mm i.d., 0.25 μm) (J&W, Folsom, CA, USA). Helium at a flow rate of 1.25 mL/min served as the carrier gas. The mass selective detector was maintained at an ion source temperature of 230 °C and a quadruple temperature of 180 °C. Electron impact (EI) mass spectra were obtained at the acceleration energy of 70 eV. About 1.0 μL aliquot of extract was injected in the split/splitless mode using an auto sampler 7683 (Agilent Technologies, Inc., Beijing, China).

In the full scan mode, fragment ions were examined over the mass range of *m/z* 40–6000. Data were acquired using the ChemStation B.02.02 software, with the integration parameters as described in Ochieng et al.^47^ with slight modifications.

### Analysis of the sterols

Mass spectral data and retention times were compared with that of cholesterol standards and reference spectra published by library-MS databases, including National Institute of Standards and Technology (NIST) 08 and 11, to identify the sterol components. The matching level of quality for the identification of the sterols was taken to be ≥ 90% with exception of a few considered above and below 70% to be traces as indicated in the table. The content of fatty acids and sterols were calculated from the relative peak area of all the detected peaks and a percentage calculated thereafter.

### Antimicrobial activity

Antimicrobial activity was carried out using the Gram positive (*B. subtilis* and Methicillin-susceptible *S. aureus* 25923) and Gram-negative (*E. coli* 25922) bacteria. A few single bacterial colonies from an overnight culture on Mueller‐Hinton Agar (MHA) were inoculated into sterile distilled water to achieve a turbidity of 0.5 McFarland ≈ 1 × 10^8^ CFU/mL as per Clinical and Laboratory Standards Institute (CLSI), by measuring the optical density (OD) = 0.132 at 630 nm.

### Inhibitory assays

The disk-diffusion assay was performed in sterile Mueller Hinton agar (MHA) medium prepared in separate sterile petri dishes; 90 mm in diameter (F&S Scientific, Nairobi, Kenya), and 25 mL was poured to each plate as described by Hudzicki^48^. From the overnight microbial cultures prepared as mentioned above, 100 μL from each bacterial species was spread uniformly using sterile beads, on separate petri dishes. Sterile 6 mm discs were placed onto each agar plate (including 2 other discs for the positive and negative control). To the disc, 20 µL of the sample solutions (10 mg/mL and 1 mg/mL for the positive control) were added, before the dishes were incubated for 24 h at 37 °C.

All the extracts were subjected to Minimum Inhibitory Concentration (MIC) and Minimum Bactericidal Concentration (MBC) against the *S. aureus*, *B. subtilis*, and *E. coli*, following published protocols with minor modification^49^. MIC assays were conducted in 96 well microtiter plates in a serial dilution, ranging from 5, 2.5, 1.25, 0.625 and 0.313 mg/mL per extract using Mueller Hinton broth (MHB). First, 40 µL of MHB was pipetted into the wells, then 40 µL of each extract at a higher concentration (i.e. 10 mg/mL for a well of 5 mg/mL), in 5% DMSO was dispensed into respective wells. Finally, 10 µL of the test bacteria in autoclaved distilled water at 1.0 × 10^8^ CFU/mL (OD = 0.132 at 630 nm) were dispensed in all the wells using a pipette, before the plates were covered with sterile lid and incubated for 24 h at 37 °C in an incubator shaker. Streptomycin (1 mg/mL) was used as the positive control, while 5% DMSO was used as the negative control. After incubation, 20 µL from wells with no turbidity were plated out on Mueller Hinton agar plate and was incubated for 24 h at 37 °C. The least concentration that showed no visible growth was taken as MBC. Triplicate experiments were conducted.

### Data analysis

The data obtained from the GC–MS was analyzed using the MSD ChemStation Data Analysis Application software equipped with Adams2, Chemecol and NIST11 database libraries. The chromatograms were illustrated using a graphical design software (Adobe illustrator CS2). One-way ANOVA statistical analysis was done using the R software version 2022.

### Ethical approval

**Institutional Review Board Statement:** The Authority to conduct the experiments and collect data was in accordance with the animal welfare regulations and granted by National Commission for Science, Technology, and Innovation (NACOSTI); Research Permit License No: NACOSTI/P/21/8303. This research also received approval from the Food Crops Research Institute where the seed specimens were collected and the Institutional Animal Care and Use Committee (IACUC) of Kenya Agricultural and Livestock Research Organization (KALRO)-Veterinary Science Research Institute (VSRI); Muguga North upon compliance with all provisions vetted under and coded: KALRO-VSRI/IACUC028/16032022. All the experiments were carried out in accordance with relevant guidelines in the method section.

## Data Availability

The datasets generated from GC–MS and analysed during the study are included in this paper.
